# Patient portal registration and healthcare utilisation in general practices in England: a longitudinal cohort study

**DOI:** 10.3399/BJGPO.2023.0106

**Published:** 2024-05-01

**Authors:** Abrar Alturkistani, Thomas Beaney, Geva Greenfield, Ceire E Costelloe

**Affiliations:** 1 Department of Primary Care and Public Health, Global Digital Health Unit, Imperial College London, London, UK; 2 National Institute for Health Research Applied Research Collaboration Northwest London, Imperial College London, London, UK; 3 Department of Primary Care and Public Health, Imperial College London, London, UK; 4 Health Informatics, Division of Clinical Studies, Institute of Cancer Research, London, UK

**Keywords:** patient portals, general practice, delivery of health care, primary healthcare, electronic health records

## Abstract

**Background:**

Patient portals introduced in most of England’s general practices since 2015 have the potential to improve healthcare efficiency. There is a paucity of information on the use of patient portals within the NHS general practices and the potential impact on healthcare utilisation.

**Aim:**

To investigate the association between patient portal registration and care utilisation (measured by the number of general practice consultations) among general practice patients.

**Design & setting:**

A longitudinal analysis using electronic health record data from the Clinical Practice Research Datalink (CPRD).

**Method:**

We analysed patients registered for patient portals (*n* = 284 666), aggregating their consultations 1 year before and 1 year after registration. We ran a multilevel negative binomial regression model to examine patient portal registration’s association with face-to-face and remote consultations.

**Results:**

Patients who registered to the portal had a small decrease in the total number of face-to-face consultations after registering to the patient portal (incidence rate ratio = 0.93, 95% confidence interval [CI] = 0.93 to 0.94). Patients who registered to the portal had an increase in the total number of remote consultations after registering to the portal (incidence rate ratio = 1.16, 95% CI = 1.15 to 1.18).

**Conclusion:**

The study found minor changes in consultation numbers post-patient portal registration, notably with an increase in remote consultations. While causality between portal registration and consultation number remains unclear, the potential link between patient portal use and healthcare utilisation warrants further investigation, especially within the NHS, where portal impacts are not well-studied. Detailed portal utilisation data could clarify this relationship.

## How this fits in

Patient portal registration and healthcare utilisation was not explored in the context of the NHS of England general practices where there is a policy-level influence to increase the uptake of patient portals. To our knowledge, this is the first study to look at patient portal registration and healthcare utilisation in the context of general practices in England. A change in the pattern of consultations after patient portal registration was observed. However, this study could not assess if the changes in healthcare utilisation outcomes were solely owing to patient portal registration. More detailed information on the use of patient portals, potentially from patient portal log files, could help clarify the mechanisms involved between patient portal registration and healthcare utilisation.

## Introduction

The NHS in England has been encouraging health information technology as part of the digital transformation plans to improve communication and access to services.^1^ Since 1 April 2015,^2^ general practices in England have been required to offer access to online services (referred to as patient portals in this study) with the main features including online appointment booking, repeat prescription ordering, and health record viewing.^3^ While there has been an increase in the promotion and use of patient portals in NHS general practice settings, there is a lack of research on the benefits of these services to patients or their associations with healthcare utilisation.

There are several explanations of how patient portal use — particularly the use of specific functionalities of patient portals, including online appointment booking, repeat prescription ordering, and online record viewing — could be associated with healthcare utilisation. One explanation comes from the exploration of how patient portals can influence patients’ information-seeking behaviour and their engagement with the healthcare system.^4^ A study that found a statistically significant increase in appointments after giving individuals’ access to clinical notes, speculated that access to information encouraged patients to inquire and request further information leading to increased number of appointment bookings.^4^ Another suggested explanation is that because patients who used patient portals are generally more likely to have long-term or chronic conditions, they are also more likely to need an increased number of appointments.^4^ One study employed a survey to measure patient activation, which was defined as the patient’s knowledge, confidence, and skills in managing their condition, and found an association between using patient portals and patient activation in the hospital setting.^5^ However, the study also used a safety display (an additional intervention) as well as patient portals and noted that patients who use patient portals may have higher patient activation at baseline.^5^ In a scoping review about older people receiving care in hospitals, patient portals were reported to increase patients' acquisition of information, enhance communication with healthcare providers, and improve trust and provision of care,^6^ all of which are factors that could be associated with healthcare utilisation. Among young adults aged 18–29 years, those who used patient portals had greater patient engagement scores (measured by commitment, informed choice, and navigation of the healthcare system) and had higher encounters with the healthcare system.^7^ Another study found that while office visits increased among patients after patient portal use, emergency department visits decreased suggesting that increased office visits may be contributing to preventive care for patients and increasing engagement with the healthcare system.^8^

Patient portals have been found to improve healthcare delivery^9^ and improve efficiency in some healthcare systems,^10^ in different settings, through improving measures such as no-show rates, reducing telephone calls, and improving patient-centred care. Patient portals have been shown to reduce no-show rates when implemented with other interventions, including appointment reminder text messages, which allow confirmation of attendance or cancellation by the patient, and appointment reminder telephone calls.^11^ Patient portal users may make fewer telephone calls to the healthcare organisation,^12^ have lower^8,12^ or no difference in visits to the emergency department visits,^13^ have fewer hospitalisations,^8^ and have increased visits to primary care or outpatient clinic visits.^8,13^ However, the evidence mentioned is from studies that were performed in different healthcare settings and with portals of different functionalities.^8^ Among patients with cancer, increased frequency of patient portal access was associated with patient-centred care delivery (where the patient’s needs, wellbeing, preferences, and understanding of their care are addressed and the patient is involved in decisions about their care),^14^ which can improve communication and the quality of care.^15^

To the authors’ best knowledge, there were no studies performed exploring patient portal registration and healthcare utilisation outcomes in the context of primary care in England. Additionally, during the coronavirus disease (COVID-19) pandemic, there was a reduction in consultations overall^16^ but also an increase in remote consultations and possibly an increase in the use of patient portals.^17^ Therefore, the use of patient portals and its influence on the healthcare system is becoming more relevant given that remote healthcare services gained popularity during this period.

In this study we aimed to investigate if there is a relationship between patient portal registration and the primary healthcare utilisation (measured by the number of general practice consultations) among general practice patients in England.

## Method

The REporting of studies Conducted using Observational Routinely-collected health Data (RECORD)^18^ checklist is used to report the methodology of the study. Data cleaning and analysis were conducted using RStudio IDE (release 2022.07.0).

### Study design and setting

This was a cohort study using patient-level data from the Clinical Practice Research Datalink (CPRD) Aurum database. The Aurum database is a collection of electronic health records from practices that use the EMIS Health electronic health record (EHR) system.^3^ The data are pseudonymised, real-world data collected through general practices in England, which includes consultations, demographic information, and diagnoses. Additionally, CPRD offers linkage to several data sources such as deprivation information. The November 2021 release of CPRD Aurum used in this study covered 19.80% of the UK population.^19^ Patients were followed-up for 12 months before patient portal registration and 12 months after patient portal registration. We selected a 12-month follow-up before and after registration to observe changes over a sufficient and reasonable timeframe. This period aligns with previous patient portal studies for better comparability.^20^ A study that used 6 months follow-up time after patient portal use mentioned that 6 months may have been too short to observe changes.^21^ A 12-month duration ensured adequate data collection while managing practicality and participant retention.

### Participants and study size

**Figure 1.**
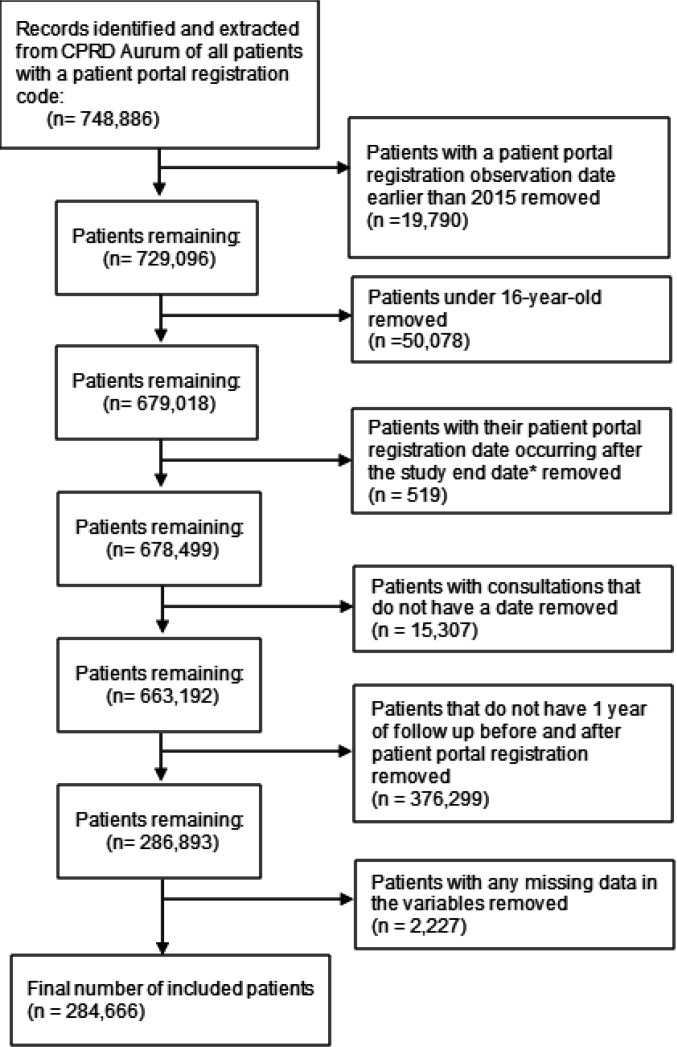
Flow diagram of study population *End date is the death date, or registration end date, or practice last collection data (whichever occurred first). CPRD = Clinical Practice Research Datalink

We first identified and extracted all patients in CPRD who had a patient portal registration code (a list of codes we developed by conducting code browser searches is available in Supplementary Box S1). All the data received from CPRD were of patients with a research acceptable flag (an indicator for having up to research standard data). Study dates varied for each patient based on their patient portal registration date. For each patient, the study start date was 1 year before the patient portal registration date and the study end date was 1 year after the patient portal date. Patients who left the study for any reason (death, leaving the practice, or end of the data collection from practice), before completing 1 year of follow-up, were excluded from the study. Study end date was different for each patient and ranged from 2 February 2016–15 August 2021. The cohort of patients received from CPRD was 748 886 and included any patient in CPRD who had a patient portal registration code in any date after 1 January 2011. We picked the date (1 January 2011) initially to make sure to include all possible patients with a patient portal registration code. We then restricted the cohort to patients registered to the portal on or after 1 January 2015 (the date when general practices in England were required to offer patient portals to patients was 1 April 2015,^2^ although many practices started to offer them the year before)^22^ (Figure 1). We then applied our inclusion criteria, which included the following: aged ≥16 years; having a patient portal registration date within the study period; having a date for every consultation (to determine how many consultations occurred before and after patient portal registration); having at least 1 year of follow-up before and after patient portal registration (Figure 1). We also excluded patients with any missing data (or indeterminate category of the gender variable) in the variables: gender and patient level English Index of Multiple Deprivation (IMD) 2019^23^ quintiles. The indeterminate gender category was removed because the number of patients in this category was very small (about 0.01% of the population). There were no missing data in the rest of the variables, and the missing data in the ethnic group variable was turned into a category called 'missing'. The final number of included patients was 284 666 (Figure 1).

Patient portals are widely available in England, and as of 30 June 2023, approximately 50% of general practice patients in England were registered to use at least one of the patient portal features, including online appointment booking, repeat prescription ordering, and viewing detailed GP records, according to GP practice-level data obtained from the patient online management information (POMI) report by NHS Digital.^24^ However, when conducting a patient-level study to investigate the associations with patient portal use in England, we encountered a limitation in the recording of patient portal use within the CPRD database. To address this limitation, we contacted CPRD at the inception of the study and sought guidance on identifying relevant codes related to patient portal use. CPRD recommended conducting a key term search in both the CPRD Aurum and CPRD Gold code browsers. We used detailed search terms such as 'online', 'portal', and 'digital' to identify any relevant codes, which were carefully recorded.

It is important to note that for our study, only codes related to having access to the medical record were considered relevant and used as a proxy for patient portal usage (Supplementary Box S1). We acknowledge that this approach represents a limitation of our study; however, these codes were the only available data at the time of conducting the research. Because patient portals in the NHS in England offer various functionalities beyond access to medical records, we cannot definitively conclude that patients without access-related codes did not register or use a patient portal. Since the use of these functionalities was not recorded in the CPRD, we could not assume that patients without access to the medical record, as identified by the codes in the Supplementary Box S1, were indeed controls. As a research team, we made the decision to adopt a research design that involved comparing patient outcomes before and after portal registration. We believe this approach was the most suitable given the available data and potential confounding variables.

### Variables and data sources

The outcome variable was the total number of GP consultations that took place. Consultations were identified by using a list of codes that indicated a consultation in the CPRD consultations files and were categorised into consultation type (either face-to- face or remote, using categorisation of consultation files supplemented with observation files, as explained in Supplementary Box S2). The list of codes for identifying consultations and their categories were adopted from the study by Foley *et al*.^25^ A detailed explanation of how each of the outcome and predictor variables were determined for the study is explained in Supplementary Box S2. Scheduled or missed appointments were not part of the outcomes of this study and the outcome only included consultations that actually took place. We separated the consultations by type of consultations (face-to-face or remote) and determined the number of consultations for each patient, we aggregated the total number of consultations to 1 year before and 1 year after patient portal registration. The main predictor variable was patient portal registration. Patient portal registration date ranged from 1 January 2015–14 August 2020. In addition to patient portal registration status, we also included: age group (based on age at registration), gender, self-reported ethnic group, long-term condition status, hearing loss, deprivation quintile, and general practice rurality, all of which are explained in detail in Supplementary Box S2. Each of the variables included in the study were chosen based on previous literature that reported that these variables could be associated with patient portal use. The variables were chosen based on previous literature and CPRD data availability, and were placed in the model to reduce potential bias from confounders. To account for the COVID-19 lockdown, we created a variable called 'follow-up time after COVID-19 lockdown', which indicated if the patient had at least 6 months of follow-up after the first COVID-19 lockdown (Supplementary Box S2).

### Statistical methods

The consultations for each patient were divided into the following two aggregate groups: those that occurred before portal registration; and those that took place afterward. Aggregate number of consultations before and after patient portal registration were identified using a binary variable (0 and 1) that represents patient portal registration. Therefore, each patient was recorded twice in the dataset to represent their total number of consultations before patient portal registration (at 0) and after patient portal registration (at 1). We ran multilevel negative binomial regression to explore the association between the total number of consultations and patient portal registration separately for face-to-face and remote consultations. The negative binomial model was chosen owing to overdispersion of the data where the variance of the number of consultations was 16 times the mean of the consultations. A multilevel model (hierarchical nesting) was applied to account for interpretability of all patients registered in a single general practice. Multilevel modelling is a type of statistical modelling method used for data that has a hierarchical or clustered structure.^26^ This means that the data are involved data subjects (for example, patients or students) that are clustered within higher level groups such as hospitals or schools (in the case of this study, it is patients clustered within general practices). It is possible to conduct modelling without taking into account the hierarchical nature of the data. However, this can result in biased results because it is likely that individuals from the same group (in this case patients from the same general practices) will be more similar to each other in characteristics compared with individuals from another group. This type of modelling is conducted in three major steps, which were followed in this study:

1. Running null model: to run the multilevel negative binomial model, we first ran a null model to explore clustering on GP level. This step determined if multilevel modelling was a suitable method for our study. In this step, we checked the interclass correlation coefficient (ICC), which is a value that ranges from 0–1, and is the value that is attributable to the differences between groups. ICC with a value of 0 indicates no clustering at the group level and a value of 1 indicates perfect clustering at group level.
2. Adding level one predictors: because the model included more than one level (patients at level 1 and GP practices at level 2), there were more than one type of predictors included and those predictors had to be included in separate steps.^27^ Therefore in step 2, we added all of the patient-level variables (level 1) to the model, which included age group, gender, ethnic group, long-term condition status, hearing loss, deprivation quintile, and the variable indicating follow-up time after the first COVID-19-related lockdown. The variables were added to the model based on what was assumed to be associated with the outcome and exposure variables.
3. Adding level two predictors: in the last step of multilevel modelling, the level two predictors are added. In our study, we only had one groups-level variable, which was the GP-level practice rurality variable. After running all of these steps, we only presented the results of the final model below.

### Secondary analysis

There were two types of consultations in our study: face-to-face and remote. Face-to-face consultations were consultations in general practices that included in-person contact with the patient, while remote consultations included telephone consultations.^25^ For secondary analysis, we included all types of consultations (face-to-face and remote) to explore the relationship between total overall consultations and patient portal registration following the same methods as above.

### Sensitivity analysis

The date of the latest available data for this study was 15 August 2021, which included the period of the COVID-19 pandemic and the lockdown that followed. Because it is likely that the number of consultations after the COVID-19 lockdown reduced or changed, we ran a sensitivity analysis excluding follow-up date further than the end of February 2020 (the first COVID-19-related lockdown in England was in March 2020)^28^ by allowing the latest patient portal registration to be on 8 February 2019. We then ran the same steps described in statistical methods subsection to explore the relationship between face-to-face consultations, remote consultations, and overall consultations (including face-to-face and remote) and patient portal registration during the period before the COVID-19 lockdown.

## Results

### Descriptive data

There were 284 666 patients in this study. Most were female (58.2%), were of the White ethnic group (70.8%), had at least one long-term condition, were in the middle (third) IMD quintile group (21.9%), and did not have a follow-up time after the COVID-19 lockdown (85.6%) (Table 1). The largest age group was that of 45–54 years (20.6%) (Table 1). The total consultations per patient over the 2-year follow-up ranged from 1 to 748, with a mean of 16.21 and a median of 11.

**Table 1.** Summary statistics of the study population (*n* = 284 666)

Patient characteristics	Overall (*n* = 284 666)
**Gender**	
Female	165 594 (58.2%)
Male	119 072 (41.8%)
**Age group, years**	
16–24	20 206 (7.1%)
25–34	38 320 (13.5%)
35–44	48 266 (17.0%)
45–54	58 554 (20.6%)
55–64	54 652 (19.2%)
65–74	42 758 (15.0%)
75–84	17 430 (6.1%)
≥85	4480 (1.6%)
**Ethnic group**	
White	201 410 (70.8%)
South Asian	19 882 (7.0%)
Black	10 736 (3.8%)
Mixed	3510 (1.2%)
Other	3879 (1.4%)
Missing	45 249 (15.9%)
**Long-term condition status**	
No	50 230 (17.6%)
Yes	234 436 (82.4%)
**Hearing loss condition**	
No	216 703 (76.1%)
Yes	67 963 (23.9%)
**IMD quintile**	
1 (least deprived)	50 886 (17.9%)
2	59 597 (20.9%)
3	62 386 (21.9%)
4	59 708 (21.0%)
5 (most deprived)	52 089 (18.3%)
**Had consultations after^a^ COVID-19 lockdown**	
No	243 646 (85.6%)
Yes	41 020 (14.4%)

^a^At least 6 months of follow-up consultation period after COVID-19 March 2020 lockdown. IMD = Index of Multiple Deprivation.

### Association of patient portal registration with face-to-face and remote consultations

Not all patients had a face-to-face or remote consultation; therefore, the number of patients included in these analyses varied. The number of patients who had a face-to-face consultation was 284 312, while the number of patients who had a remote consultation was 59 298. The incidence rate ratio for total face-to-face consultations was 0.93 (95% confidence interval [CI] = 0.93 to 0.94), which means there was a 7.0% decrease in the rate of consultations 1 year after registering to the portal compared with 1 year before registering to the portal (Table 2). On the other hand, the rate ratio for total remote consultations was 1.16 (95% CI = 1.15 to 1.18), which means there was a 16.0% increase in the rate of consultations 1 year after registering to the portal compared with 1 year before registering to the portal (Table 3).

**Table 2.** Results of the fully adjusted multilevel negative binomial regression of patient portal registration and other patient and GP-level characteristics on face-to-face consultations (level 1, *n* = 568 624 observations of 284 312 patients; level 2, *n* = 897 general practices)

Predictors	Incidence rate ratios	95% CI	*P* value
**Registration to the patient portal (ref = No) **			
Yes	0.93	(0.93 to 0.94)	<0.001*
Gender: Male (ref = Female)	0.80	(0.804 to 0.796)	<0.001*
Age group, years (ref: 45–54)			
16–24	0.89	(0.88 to 0.90)	<0.001*
25–34	0.98	(0.97 to 0.99)	<0.001*
35–44	0.96	(0.95 to 0.96)	<0.001*
55–64	1.08	(1.08 to 1.09)	<0.001*
65–74	1.26	(1.26 to 1.27)	<0.001*
75–84	1.56	(1.55 to 1.58)	<0.001*
≥85	1.61	(1.58 to 1.64)	<0.001*
Ethnic group (ref: White)			
Asian	1.16	(1.15 to 1.17)	<0.001*
Black	1.08	(1.07 to 1.10)	<0.001*
Mixed	1.04	(1.02 to 1.06)	<0.001*
Other	0.99	(0.97 to 1.01)	0.213
Missing	0.91	(0.90 to 0.92)	<0.001*
Hearing loss: Yes (ref = No)	1.26	(1.25 to 1.27)	<0.001*
Long-term condition: Yes (ref = No)	1.89	(1.87 to 1.90)	<0.001*
Consultations during COVID-19 lockdown: Yes (ref = No)	0.78	(0.77 to 0.78)	<0.001*
Deprivation quintile (ref: 5, Most deprived)			
1, least deprived	0.95	(0.94 to 0.96)	<0.001*
2	0.91	(0.90 to 0.92)	<0.001*
3	0.93	(0.92 to 0.93)	<0.001*
4	0.96	(0.95 to 0.96)	<0.001*
General practice rurality: Rural (ref = Urban)	1.02	(0.95 to 1.10)	0.570
Interclass correlation coefficient	0.18		

Bold and asterisked = statistically significant at the *P*<0.05 level.

**Table 3.** Results of the fully adjusted multilevel negative binomial regression of patient portal registration and other patient and GP-level characteristics on remote only consultations (level 1, *n* = 118 596 observations of 59 298 patients; level 2, *n* = 667 general practices)

	Remote consultations only			
**Predictors**	**Incidence rate ratios**	**SE**	**95% CI**	***P*** **value**
Registration to the patient portal: Yes (ref = No)	1.16	0.01	(1.15 to 1.18)	<0.001*
Gender: Male (ref = Female)	0.90	0.01	(0.89 to 0.92)	<0.001*
Age group (ref: 45–54)				
16–24	1.04	0.02	(1.00 to 1.07)	0.040*
25–34	1.05	0.01	(1.02 to 1.07)	<0.001*
35–44	1.02	0.01	(0.99 to 1.04)	0.203
55–64	1.02	0.01	(0.99 to 1.04)	0.184
65–74	1.03	0.01	(1.01 to 1.05)	0.016*
75–84	1.21	0.02	(1.18 to 1.25)	<0.001*
≥85	1.43	0.03	(1.37 to 1.49)	<0.001*
Ethnic group (ref: White)				
Asian	1.07	0.02	(1.03 to 1.10)	<0.001*
Black	1.05	0.02	(1.01 to 1.08)	0.012*
Mixed	1.03	0.03	(0.98 to 1.09)	0.246
Other	0.96	0.03	(0.90 to 1.02)	0.146
Missing	0.98	0.01	(0.96 to 1.00)	0.054*
Hearing loss: Yes (ref = No)	1.12	0.01	(1.10 to 1.14)	<0.001*
Long-term condition: Yes (ref = No)	1.37	0.02	(1.33 to 1.40)	<0.001*
Consultations during COVID-19 lockdown: Yes (ref = No)	1.16	0.01	(1.13 to 1.18)	<0.001*
Deprivation quintile (ref: 5, Most deprived)				
1, Least deprived	0.97	0.01	(0.95 to 1.00)	0.027*
2	0.94	0.01	(0.92 to 0.96)	<0.001*
3	0.96	0.01	(0.94 to 0.99)	0.003*
4	0.98	0.01	(0.96 to 1.00)	0.056*
General practice rurality: Rural (ref = Urban)	1.03	0.05	(0.94 to 1.13)	0.496
Interclass correlation coefficient	0.11			

Bold and asterisked = statistically significant at the *P*<0.05 level. SE = stadard error.

### Association of patient portal registration with overall GP consultations

In the fully adjusted model, the rate ratio of overall consultations (including face-to-face and remote) after registering to the patient portal was 0.94 (95% CI = 0.94 to 0.95), which means there was about 6.0% reduction in total consultations 1 year after registering to the portal compared with 1 year before registering to the portal (Supplementary Table S3).

### Results of the sensitivity analysis

The results of the sensitivity analysis, which excluded follow-up date further than the first COVID-19 lockdown in England, of the face-to-face consultations and remote consultations are in Supplementary Table S4. The rate ratio of total face-to-face consultations after registration was 0.99 (95% CI = 0.98 to 0.99) in the period before the COVID-19 lockdown. The rate ratio of total remote consultations after registration was 1.03 (95% CI = 1.01 to 1.05) in the period before the COVID-19 lockdown.

The number of patients in the sensitivity analysis of overall consultations was 215 433 (75.7% of the total sample). The rate ratio of total consultations after registration was 0.99 (95% CI = 0.98 to 0.99) (Supplementary Table S5), indicating no difference in the number of total consultations after registering to the patient portal in the period before the COVID-19 lockdown.

## Discussion

### Summary

The study found a small, but statistically significant decrease in face-to-face consultations, and an increase in remote consultations, after patient portal registration, which neutralised in the sensitivity analysis excluding the period after the first COVID-19 lockdown in England. When including all types of consultations (face-to-face and remote), the study found a small reduction in consultations after patient portal registration. However, when excluding the period post-COVID-19 lockdown, there was no difference in the overall consultations after registering to the portal. Only the face-to-face consultations maintained an increase after removing the period post-COVID-19 lockdown, but the increase was very small.

### Strengths and limitations

To the best of our knowledge, this is the first study to use CPRD to explore patient portal registration and its relationship with consultations in England. Compared with the complete CPRD Aurum population in September 2018,^29^ our sample had slightly higher percentage of females, and had similar percentages in terms of the English regions the patients are from. We could not compare our study population to the CPRD Aurum population in terms of age because our population only included patients aged ≥16 years and the complete cohort includes patients aged <16 years.^29^

Using patients’ access or registration to the online record as an indicator for patient portal use can lead to bias and overestimation of patients using the portal as the adoption of the portal does not indicate continuous use or long-term use.^11,30,31^ We could not compare our patient cohort with a group that does not have a patient portal registration because there is no guarantee that the patients who we did not identify through the codes were not registered to a patient portal. According to the POMI data,^24^ at the end of August 2021 there were at least 40% of patient portal users in all NHS general practices, which is not reflected in the CPRD data (there were 40 265 295 patients in the CPRD Aurum November 2021 release and only 1.9% [*n* = 748 886] had a patient portal registration code). This may be because patient portal registration and use is not well recorded in CPRD.

In this study, disease severity may have been an important factor in determining the number of consultations. Owing to the large number of conditions considered in our study (211), it was not feasible to directly account for disease severity. Additionally, studies that use CPRD and take into account disease severity generally focus on one or two diseases.^32^ Limiting the study to only one or two disease would have limited the study sample and conclusions. As a result, the severity of the 211 conditions included in our analysis was not individually assessed.

### Comparison with existing literature

Pre-existing evidence for the relationship between patient portal use and consultations is not clear. Previous studies did find an increase in consultations among patients using patient portals.^8,12,33^ However, the studies differed in their patient populations and in their definitions of the consultations. One reason why this study found contrasting associations between patient portal use and consultations may be owing to not having detailed information about patient portal use. Studies show that registration to the patient portal does not guarantee active use of the portal (for example, using the portal to book appointments or check test results).^34^ Detailed information on patient portal use, such as messaging the healthcare provider or ordering repeat prescription or information from patient portal log files, could reveal clearer associations with the number of consultations.

### Implications for research and practice

We found small differences in the number of consultations in this study and we cannot completely conclude that the decrease or increase in consultations is owing to patient portal registration. A notable difference was in the result of analyses for remote consultations both including all of the study period and the sensitivity analysis period, which resulted in a rate ratio of 1.16 in the period including the COVID-19 lockdowns in England compared with 1.03 in the period before the lockdown only. This indicated that there has been a notable increase in remote consultations after the COVID-19 lockdown, although this is expected. This aligns with previous research that observed an increase in telephone or remote consultations during and after the pandemic.^17,35,36^ The changes were largely driven by the NHS’s efforts to control the spread of the virus and protect vulnerable populations. Although we cannot exclusively attribute the increased rate in consultations to the registration to the portal, it is noteworthy to mention that the post-COVID-19 period was controlled for in the analysis. In addition, the knowledge that remote consultations increased after the COVID-19 pandemic makes it more important to further explore technologies that allow patients to remotely connect to the healthcare system such as the patient portals. Patient portals and their association with healthcare system utilisation remain understudied in the context of the NHS, and further studies and better data are needed to explore these mechanisms.

## Data

Data used in this study cannot be made publicly available as it contains sensitive patient information. However, it can be accessed through an application to the Clinical Practice Research Datalink (https://www.cprd.com/) subject to a review process.
